# Historical Process, Status and Future Development of Pharmacovigilance Systems in Chinese Medicine

**DOI:** 10.3390/pharmacy13040090

**Published:** 2025-06-26

**Authors:** Xue Wang, Yinfeng Wang, Xiaomeng Zhang, Bing Zhang, Zhijian Lin

**Affiliations:** 1 Department of Clinical Chinese Pharmacy, School of Chinese Pharmacy, Beijing University of Chinese Medicine, Beijing 100029, China; wangxue20220222@163.com (X.W.); wangyinfeng24@163.com (Y.W.); zhangxm0320@163.com (X.Z.); zhangb@bucm.edu.cn (B.Z.); 2 Research Center for Pharmacovigilance and Rational Use of Chinese Medicine, Beijing University of Chinese Medicine, Beijing 100029, China

**Keywords:** pharmacovigilance, adverse drugs reaction, drug regulation, policy, management

## Abstract

The definition of pharmacovigilance was first proposed in the 1970s to safeguard public health and the safety of drug use, and to improve the quality of life of patients. China’s traditional medicine has also always contained the idea of vigilance for the safe use of medicines in the process of development. In this article, we will discuss the origin and development of the idea of pharmacovigilance in China, the establishment of a realistic system, and the current status of pharmacovigilance construction, and look forward to the development of pharmacovigilance in the future.

## 1. Introduction

As early as 1968, the World Health Organization (WHO) had launched an international drug monitoring program, and in 1974 French scientists first introduced the concept of pharmacovigilance “to monitor, prevent, and be ready to respond to the possible hazards of drugs” [1]. In 1992, the pharmacoepidemiologist Begaud [2] provided a clear definition of pharmacovigilance, stating that all methods of preventing and detecting adverse drug reactions should not be limited to post-marketing drugs, but should also include pre-marketing clinical trials and even pre-clinical trial studies. It was not until 2002 that the WHO updated and refined the definition of pharmacovigilance to “the science and activities to detect, evaluate, understand and prevent adverse drug effects or any other drug-related problems” [3].

Pharmacovigilance is dedicated to safeguarding public health and the safety of drug use and is essential in assessing drug side effects and is an important support for the pharmaceutical industry. As a member of the International Pharmaceutical Testing Program, China has been monitoring adverse drug reactions since the 1980s and has gradually introduced the concept of pharmacovigilance. China has started to actively explore the pharmacovigilance system and related laws and regulations in line with China’s national conditions and has now applied pharmacovigilance theory and practice to the whole life cycle of drugs, covering the safety regulation of chemical drugs, Chinese and ethnic medicines, biological products, blood products, vaccines and medical devices [4].

Until 2019, the formal establishment of the pharmacovigilance system, together with the drug traceability system and drug listing licensee system, constituted the basic system of drug management in China [5]. Meanwhile, the promulgation of the Pharmacovigilance Quality Management Standard by National Medical Products Administration(NMPA) took place in 2021 [6]. Moreover, the development of pharmacovigilance has been pushed to a climax. However, today, with COVID-19, China’s pharmacovigilance system has ushered in new opportunities and challenges.

This paper systematically compares the origin and development of traditional Chinese medicine pharmacovigilance ideas and the Chinese pharmacovigilance system and regulatory model, discusses the evolution of the Chinese pharmacovigilance system from the perspective of historical processes, analyzes the current problems and challenges faced, and clarifies the future development direction of Chinese pharmacovigilance, with a view to establishing a sustainable pharmacovigilance path with Chinese characteristics.

## 2. The Idea of Pharmacovigilance in Traditional Chinese Medicine

In the process of practice, Chinese traditional medicine has always embodied a vigilant approach to safe drug use, and formed an approach to drug vigilance in the concept of “knowing poison–using poison–preventing poison–solving poison”, with “poison” as its core [7].

The knowledge of the safety of Chinese medicine can be traced back to the ancient times, such as in *Huainanzi—Xiuwu Xun*, which says, “In one day, one encounters seventy poisons” [8]. In the Zhou Dynasty, there is a record that “the physician is in charge of the administration of medicine and gathers poisons for medical practice” in the Zhou Li–Tian Guan–Tsukazai [9]. The germ of traditional Chinese cautionary thinking with regard to drugs began with The *Yellow Emperor’s Canon Internal Medicine*, the founding work of Chinese medicine theory during the Qin and Han dynasties of the Warring States period, which recorded and discussed the possible harm of drugs to the human body, and for the first time classified drugs into two types: poisonous and non-poisonous [10]. It is the first time that drugs were classified as toxic or non-toxic.

In the Han Dynasty, the idea of drug vigilance was initially formed, such as in *Shen Nong’s Herbal*’s [11] division of drugs into toxic and non-toxic: the 365 recorded drugs were divided into three classes (Upper, Middle, Lower) according to their functions and into toxic and non-toxic. It was stated that toxic drugs should not be used for a long time, and that when toxic drugs were used in treatment, the lowest dose should be used first; it was stated that for strong drugs used for medication, when the disease has largely gone, we must stop the drug use, or use other drugs to regulate; and other vigilance ideas were put forward for the first time, which laid a solid foundation for the establishment of the drug vigilance system of traditional Chinese medicine. In the Eastern Han Dynasty, the medical classic *Treatise on Cold-Induced and Miscellaneous Diseases and Synopsis of Prescriptions of the Golden Chamber* recorded and elaborated on the principles of dietary contraindications and drug dose control after taking drugs. *Miscellaneous Records of Famous Physicians* even, for the first time, classified toxic drugs into three grades—major, toxic and minor—marking the emergence of the idea of grading the toxicity of Chinese medicine [12].

During the Wei and Jin dynasties, people’s awareness of drug vigilance was further enhanced and the traditional theoretical framework of drug vigilance in Chinese medicine was basically formed. For example, in the Eastern Jin Dynasty, *A Handbook of Prescriptions for Emergencies, Volume 7*, is a special chapter on the relief of chest congestion caused by overdose, which is the earliest work in China to set up a special chapter on the method of detoxification of Chinese medicine and laid the foundation of the idea of detoxification of Chinese medicine [12].

During the Southern and Northern Dynasties, a new breakthrough was made in the development of the idea of drug caution, as shown in the publication of the *Collection of Notes on the Materia Medica*, which for the first time systematically compiled drug cautions such as “fear of evil and contraindication” and “contraindication to taking medicine and food”, and set them out in a special preface. It also further elaborated on the principle of the dose of poisonous drugs, saying that when using poisonous drugs to treat diseases, specific analysis should be made on a case-by-case basis, and the size of the dose should be determined by the amount of poison contained in the drug or the toxicity of the drug, and not be generalized [12].

During the Sui, Tang and Five Dynasties, thought on pharmacovigilance was further deepened and developed. The adverse reactions to toxic and non-toxic drugs were increasingly recorded, not only in relation to the drug itself, dosage regimen, decoction method, concoction processing, etc., but also in *A Supplement to the Compendium of Materia Medica*, which summarized the adverse reactions to each type of drug due to poison [13]. Sun Simiao, the king of medicine, devoted a special section to the relief of drug poisoning in the *Invaluable Prescriptions for Emergencies and Supplement to Invaluable Prescriptions*.

During the Song, Jin and Yuan dynasties, the idea of drug vigilance was further developed, and the traditional drug vigilance framework was further improved. For example, Tang Shenwei’s *Classic Classified Materia Medica for Emergency* of the Song Dynasty inherited and developed the discussion of drug vigilance from the previous *Materia Medica*, expanding the contents of “fear of evil and contraindication of table” and “contraindication of taking medicine and food”. Kou Zongshi emphasized that the dosage of poisons should be determined according to the specific conditions of the patient and the disease. In the Southern Song Dynasty, Zhu Duanzhang’s *A Precious Manual of Obstetrics for Home Use* contained the first song on pregnancy contraindications [14]. In the Southern Song Dynasty, Wang Huaiyin’s *Taiping Shenghui Fang–Vol. 39—Prescriptions for Poisoning of Various Medicines* and Zhao Ji’s *Complete Record of Holy Benevolence—Miscellaneous Therapies* contain insightful explanations of the development of the idea of drug caution in the Song Dynasty. In the Jin-Yuan period, the core elements of the theory of contraindications of Chinese medicine were clearly put forward, namely “18 contraindications” and “19 fears” [15].

During the Ming and Qing dynasties, traditional drug cautionary thinking expanded and enriched. For example, Li Shizhen’s *Compendium of Materia Medic* reviewed the idea of drug vigilance in the materia medica of the past generations, added a large number of toxic drugs, divided 361 kinds of poisonous drugs into four levels according to their toxicity—namely, major poison, poisonous, minor poison and slightly poisonous—and set up a special item of “poisonous herbs” for the first time to contain 47 kinds of toxic herbs [16]. In the Qing Dynasty, Wang Ang’s *The Materia Medica Easy to Read* was more detailed in the classification of drug toxicity. In the late Qing Dynasty, Ling Huan, a famous doctor, wrote *The Harmful Effects of Materia Medica* [17], which is a work that concentrates the essence of China’s traditional pharmacovigilance thinking and further develops and enriches the idea of pharmacovigilance [18]. The above can be summarized in Table 1. 

The traditional pharmacovigilance of traditional Chinese medicine is gradually developed by successive generations of pharmacists in practice and inheritance, and is the accumulation of important clinical medication experience, which plays an important role in ensuring the safe use of traditional Chinese medicine. By combining the relevant records on the safe use of medication and pharmacovigilance in the writings of various dynasties, it can be found that it involves a number of aspects: ① the toxicity rating of traditional Chinese medicine is constantly being developed; ② records on how to use drugs safely, including the dosage, duration of treatment, dietary taboos, contraindications, etc.; ③ adverse drug reactions and toxic effects are recorded; and ④ recovery after the occurrence of toxic reactions is also relevant. The “knowledge of poison-poison prevention-poison-detoxification” is embedded and continues to develop, forming the prototype of Chinese medicine pharmacovigilance, contributing to the continuous future improvement of pharmacovigilance ideas, and laying the foundation for the establishment of a pharmacovigilance system.

## 3. History of China’s Pharmacovigilance System and Practice

With the gradual start of the modern pharmaceutical industry in 1949, China began to establish a system for collecting reports of adverse drug reactions to penicillin in some areas. In 1988, pilot work on ADR monitoring was announced in 14 hospitals in Beijing, Shanghai and Guangzhou. In 1989, the former Ministry of Health established an Adverse Drug Reaction (ADR) monitoring center in the China Biological Products Testing and Certification Institute. Ten ADR testing centers were established between 1990 and 1997. In 1998, China joined the WHO National Drug Surveillance Cooperation Program and became the 68th member country. In 1999, the State Drug Administration Adverse Drug Reaction Monitoring Center was established, and the Administrative Measures for Adverse Drug Reaction Monitoring (for Trial Implementation) were promulgated. In 2000, the first national ADR monitoring work conference was successfully held. And in 2001, the Drug Administration Law provided for the implementation of the ADR reporting system [19]. The legal system of ADR reporting and the monitoring network have taken shape.

In 2002, the European Union issued an important document on pharmacovigilance, providing a definition of pharmacovigilance and guiding member states to carry out pharmacovigilance activities. China introduced the concept of pharmacovigilance in 2004, formally put the national adverse drug reaction monitoring system online, and issued and implemented the Administrative Measures for Adverse Drug Reaction Reporting and Monitoring. In 2007, an annual working meeting system for provincial directors was established. The preparation of the annual report for national adverse drug reaction monitoring began in 2009. Administrative Measures for Adverse Drug Reaction Monitoring and Management were implemented in 2011. China’s pharmacovigilance industry entered a period of quality.

In 2012, the EU released the Good Pharmacovigilance Practice guidelines, which updated the regulatory framework. In 2013, the Food and Drug Administration (FDA) was officially licensed, and the regulatory system entered a new phase. In 2017, the FDA joined the International Coordination Committee for the Registration of Pharmaceutical Products for Human Use (ICH) to translate the relevant guidelines for implementation. In 2018, the Food and Drug Administration (FDA) issued the Announcement on Direct Reporting of Adverse Reactions by Pharmaceutical Marketing Licensees, which clarified the requirements for marketing licensees (MAs) to report adverse reactions [20]. Based on this, the adverse reaction reporting system began to shift to a pharmacovigilance system, which laid the foundation for the establishment of a pharmacovigilance system.

In 2019, the country officially implemented the Drug Administration Law of the People’s Republic of China (Drug Administration Law) [21], a new milestone in the field of drug regulation, marking the elevation of pharmacovigilance to a drug regulatory system in China, and in 2021, we released and implemented the Pharmacovigilance Quality Management Code to regulate and guide the pharmacovigilance activities of drug marketing licensees and drug registration applicants, with the groundbreaking addition of a pre-marketing pharmacovigilance module that emphasizes risk management throughout the life cycle of drugs. In April 2022, the “Guidelines for Pharmacovigilance Inspection” was implemented, guiding drug regulatory authorities to conduct pharmacovigilance activities in a scientific and standardized manner [22]. In April 2022, the Pharmacovigilance Inspection Guidelines were implemented to guide drug regulatory authorities to carry out pharmacovigilance inspections in a scientific and standardized manner.

In February 2023, the State Drug Administration (SDA) issued the Special Provisions on the Registration and Management of Traditional Chinese Medicines, which further refined the requirements related to the development of traditional Chinese medicines, and strengthened the management of the development and registration of new traditional Chinese medicines. It has comprehensively and systematically constructed a registration management system for Chinese medicines, and has made every effort to promote the modernization of Chinese-style drug supervision. 2023 On September 2023, it issued Measures for the Supervision and Administration of the Quality of Drug Operation and Use, which strengthened the quality management responsibilities of holders of listed licenses for medicines, and enterprises, and refined their requirements for the management of personnel involved in the purchase and sale of medicines.

**Figure 1:**
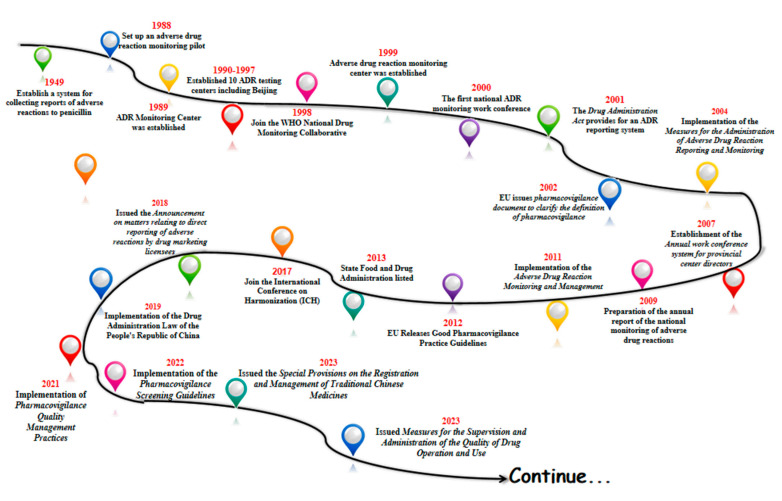
Major milestones in China’s drug administration from 1949 in China.

Going through the history of the development of China’s pharmacovigilance, one can see that after the founding of the country and with the start of industrial pharmaceuticals, the state began to strengthen the attention to adverse drug reactions, from only focusing on some areas to establishing an adverse reaction collection system in some hospitals (Figure 1). Furthermore, it carried out pilot studies monitoring adverse drug reactions, formally established of the Adverse Drug Reaction Monitoring Center of the State Drug Administration, promulgated the relevant documents for the monitoring of adverse drug reactions, and formally implemented the “Law of the People’s Republic of China on the Management of Pharmaceuticals”, which can be seen in the whole course of the development of the system of pharmacovigilance in our country. This can be seen from the beginning to the pilot studies to the implementation of the whole course of the development of the system, and at present we are also in a continuous process of improvement and progress.

## 4. China’s Pharmacovigilance Regulatory Status

China’s pharmaceutical industry has developed rapidly since the founding of the country, experiencing a change from the API era (1990–2012) to the generic era (2001–2018), to the innovative drug era (2019–present). Since the 1950s, the state started to establish ADR monitoring institutions in various regions for ADR reporting, and the development process of ADR monitoring was stalled to some extent for various reasons, such as an insufficient monitoring scale, the high rate of ADR underreporting, low social motivation, and small scope of alerting activities. The occurrence of aristolochic acid nephrotoxicity in Belgium in the late 1990s (“aristolochic acid nephropathy”) has further aroused worldwide concern about the safety of TCM [23]. The subsequent “Gentian and Liver Pill Incident” brought Chinese medicine to the limelight and completely contributed to the establishment of China’s adverse drug reaction monitoring system. The adverse reactions that occurred in the early 20th century, such as the significant increase in the number of adverse reactions reported for ichthyosanthus injection between 2003 and 2006 [24], and the 2008 incident of prickly pine injection due to drug contamination [25], accelerated the improvement of legislation and safety alert monitoring of adverse reactions in China.

In response, the State issued the Measures for the Administration of Adverse Drug Reaction Reporting and Monitoring in 2011 [26], which upholds the principle of “suspicion is reported”, strengthens post-marketing supervision of drugs, standardizes the reporting of adverse drug reactions, and controls drug risks in a timely and effective manner. The Measures for Drug Registration and Management issued in 2014 and the Drug Listing Permit Holder System issued in 2016 enacted further drug reform and movement towards the establishment of a scientific, effective and sound drug regulatory system. In 2017, we joined the ICH to align with international pharmacovigilance, and the national pharmacovigilance system became clearer. In 2019, the Drug Administration Law [27] was promulgated, and we officially entered a new period of vigilance.

In the revised Drug Administration Law in 2021, the national pharmacovigilance legal system had been formed. It clarifies the responsibility of medical institutions and drug marketing licensees to report adverse reactions, and emphasizes in Article 81 that “drug marketing licensees, drug manufacturers, drug business enterprises and medical institutions shall regularly examine the quality, efficacy and adverse reactions of drugs produced, operated and used by their own units”, actively mobilize social resources related to pharmacovigilance activities, and expand the scope of pharmacovigilance monitoring. At the same time, the decree strengthens the management of drug development stages, post-marketing supervision, and drug supply assurance, and refines the management of safety information monitoring and reporting; risk assessment; risk control and communication; post-marketing safety studies; pharmacovigilance-related document and record management, throughout the entire process of drug development; approval and marketing supervision; the whole-life-cycle management of drugs; and the whole chain of risk control. In Article 16 of the revised Drug Administration Law of 2021, it was proposed to “establish and improve the technical evaluation system in line with the characteristics of Chinese medicine”, which opened a new era of pharmacovigilance for Chinese medicine.

China’s pharmacovigilance regulatory system has undergone a transformation from adverse reaction monitoring to pharmacovigilance, and the system took shape at the beginning of this century. At the same time, China actively communicates with the WHO, Uppsala Testing Center, and other monitoring institutions in the field of pharmacovigilance. Since joining the ICH in 2017, it has gradually adopted internationally accepted pharmacovigilance standards and has actively drawn on the pharmacovigilance experience of the EU, Japan, the United States, Canada, and other countries to attach importance to international convergence, gradually forming a pharmacovigilance quality that dovetails with the international standard.

## 5. Challenges, Opportunities and Prospects

Today, with the increasing concern about drug safety, it is imperative to build a good pharmacovigilance system in China. From the ancient period of the knowledge of “poison”, through the gradual development of the traditional thought of safe drug use by successive generations of doctors, and the integration with the modern thought of pharmacovigilance, the current pharmacovigilance system in China has been formed, completing the transformation from the understanding of toxicity to the monitoring of adverse reactions to the current thought of pharmacovigilance, in addition to the active communication and exchange with the international community. In addition, the active communication and exchange with the international community has broadened our vision and made us fully aware of the deficiencies and challenges of the current pharmacovigilance system in China, and has enabled us to learn from international experience so that we can improve the domestic system and accelerate communication with the international community.

The following are challenges facing China’s pharmacovigilance system:

Challenge 1: Cultivation management and quality supervision of Chinese herbal medicines. As a unique medicinal treasure in China, Chinese herbal medicines occupy an important position in the domestic drug market. With the worldwide application of Chinese medicine in recent years, the safety of Chinese medicine has become an increasing concern, and the pharmacovigilance of Chinese medicine is facing great challenges. Compared to chemical drugs, there are more factors affecting the safety of Chinese medicine, and the aspects of risk prevention and control are more complicated. Compared with chemical drugs, Chinese herbal medicines are mainly from nature, and there may be quality and safety risks in every step of the process, from the field to clinical application [28]. The quality and safety risks may exist at every step from field to clinical application. There is no doubt that Chinese medicine poses a greater challenge to its regulation. Regarding the management of planting, China is still facing problems in the planting of Chinese herbal medicines, which directly affects the quality and subsequent clinical effects of Chinese herbal medicines. On one hand, there is a lack of promotion of good seeds, and on the other hand, there is a lack of upgrading and updating planting techniques. The backwardness of planting management technology and non-scientific planting lead to the inconsistent quality of herbal growth, which seriously affects the quality and subsequent efficacy of herbal medicine [29]. In addition to planting, there are also quality and safety risks caused by confusion of varieties, improper processing and preparation, artificial adulteration, and foreign harmful substances such as pesticide residues and microbial contamination; there are many variables such as extraction, refining, concentration, drying, molding and packaging [28]. Therefore, strengthening the management of the cultivation of Chinese herbal medicines and the subsequent quality control of all aspects of the processing of Chinese herbal medicines has become a major challenge in improving the pharmacovigilance system in China.

Challenge 2: The scope of pharmacovigilance is not comprehensive and its expression is not standardized. The concept of pharmacovigilance has been around for decades, and the World Health Organization (WHO) defines pharmacovigilance as “the science and activity of detecting, evaluating, understanding, and preventing adverse drug reactions or any other drug-related problems” [30].

On 1 December 2019, the newly revised Drug Administration Law came into force, which included the pharmacovigilance system [6]. The law proposes a new system, a new way to strengthen drug risk management and full control. The revised Drug Administration Law, Article 12, paragraph 2, states that “the State establishes a pharmacovigilance system to monitor, detect, evaluate and control adverse drug reactions and other harmful reactions related to the use of drugs.” However, there is no direct definition for pharmacovigilance [31]. At present, China’s regulatory focus on the pharmacovigilance system is still on adverse drug reactions and adverse events, while pharmacovigilance is concerned with “adverse drug reactions and other possible harmful reactions related to drugs” [32]. Therefore, the pharmacovigilance system is a comprehensive drug safety regulatory system covering the whole life cycle of drugs, not only the monitoring and reporting of adverse drug reactions, but also the misuse, abuse, drug–drug interactions, lack of efficacy and other drug-related adverse events [33]. The scope of pharmacovigilance detection should be appropriately expanded, and the concept and expression of pharmacovigilance should be standardized.

Challenge 3: Pharmacovigilance regulation on a large scale. With a population of over 1.4 billion, China is the most populous country in the world. This huge population size makes its drug use far more than other countries. The huge scale of drug use has led to varying feedback from the people after drug use, with frequent cases of misuse, misapplication, and irrational use. During the one-year period of 2020 reported by Vigibase, China accounted for 36% of the total number of reported individual safety reports (ICSRs), making it the second-largest country after the United States. The large population size has led to increased difficulty in monitoring adverse events and the emergence of many factors that affect the safety of drugs throughout their life cycle, thereby slowing the process of drug regulation.

Challenge 4: Chinese medicine drug vigilance regulation difficulties, Chinese medicine, ethnic medicine, and other full life cycle vigilances are difficult to reflect. Pharmacovigilance activities are mainly based on pharmaceuticals, including traditional Chinese medicine, chemical drugs, and biological products. The time frame of pharmacovigilance covers the whole life cycle of drugs from pre-marketing to post-marketing. Chemical and biological drugs already have good international regulatory experience, such as the sentinel program implemented in the United States to actively monitor adverse drug reactions, using a combination of ADR passive monitoring and active monitoring to reduce the occurrence of adverse drug reactions. Compared with Western countries, China has also established a legal framework for pharmacovigilance that covers the entire life cycle of drugs. Meanwhile, as the world’s largest producer and exporter of APIs, China’s current pharmacovigilance system is not fully suitable for the regulation of Chinese medicine, ethnomedicine, botanicals, etc. Whole-life-cycle vigilance in Chinese medicine needs to be urgently highlighted. Although the percentage of ADRs for TCM has been relatively low in recent years, and although there are many quality-control and vigilance procedures for TCM before marketing, post-marketing adverse reaction reports are limited, and TCM manufacturers have insufficient research initiatives and low participation in post-marketing vigilance activities for TCM, such as post-marketing clinical safety evaluations of TCM and improvements of TCM manuals, failing to form an efficient regulatory mechanism. Moreover, due to the complexity of traditional drugs such as those used in Chinese medicine and natural drugs, the specificity of clinical application, the correlation of “dose-adverse reactions”, and the prevalence of combined use of Chinese and Western medicines, there are many difficulties in the pharmacovigilance activities of Chinese medicine.

Challenge 5: Uneven regional development and difficulty in carrying out pharmacovigilance in remote areas. Regional differences and unbalanced development are the basic national conditions of China, and there are still large gaps in economic development in the east, middle and west, and the problem of unbalanced and insufficient development in urban and rural areas is still prominent, which is the general root of many problems in China at this stage [34]. One of the difficulties seen in people’s lives is how it is “difficult and expensive to see a doctor”, while there is still a shortage of medicine and out-of-date medical conditions in rural primary medical institutions. Under the conditions of drug shortages and poor medical effects, it is even more difficult to carry out pharmacovigilance, but it is also more important. Reducing the gap between urban and rural medical care and improving rural medical conditions have also become major challenges for pharmacovigilance. While trying to meet people’s medical needs, strengthening the awareness of rational drug use, collecting information on drug reactions, and reducing the occurrence of drug reactions have also become major challenges for pharmacovigilance work.

Challenge 6: E-commerce transactions and telemedicine supervision are challenging. With the rapid development of technology, the great influence of the Internet has gradually penetrated into the medical industry. This is seen with the development of the combination of “Internet + medical health” and telemedicine, and online platforms for the sale of drugs gradually entering people’s awareness, this because of its convenience, its use becoming faster with an increasing number of medical patients and consumers. However, regulation has gradually become a major problem in the development of Internet medicine.

The seeming convenience of drug sales under the surface of e-commerce transactions faces huge risks and challenges; how to regulate and how to carry out pharmacovigilance is currently facing a major problem. For example, problems include the following: whether Internet sales of drugs have legal sale qualifications; whether there is the use of the Internet to exaggerate or even use false propaganda and thus enact the sale of fake or substandard drugs; how to carry out publicity for drug information and safe drug knowledge for online drug purchases; how to recall the occurrence of adverse reactions or adverse events of drugs sold online; how to improve the after-sale service of online drug purchase [35]. How can the after-sale service of online drug purchases be improved? These potential problems are all related to how to carry out pharmacovigilance work in today’s society where drugs are widely traded through e-commerce.

Similarly, the same problem exists with Internet-based telemedicine for drug purchases. As a new medical model that integrates the fields of medicine and communication [36], telemedicine also brings convenience to people and promotes the development of superior medical resources and the accessibility of primary care; however, it also faces problems and challenges. The implementation of reasonable and efficient supervision, collection and protection of patient information and privacy, providing reasonable advice on medication use, timely post-medication visits, and adverse reaction monitoring are also major challenges for pharmacovigilance work.

The following are suggestions and prospects:

Firstly, we should broaden the scope of pharmacovigilance, standardize its formulation and scientifically grasp its meaning. Currently, as people’s standards of living improves and medical care evolves, the pursuit of life and living is gradually increasing. In order to improve the quality of people’s lives, the importance of pharmacovigilance is increasingly emphasized worldwide. The World Health Organization has announced that the scope of pharmacovigilance is expanding due to globalization, consumerism, the explosion of free trade, international exchange, and the increasing use of the Internet [37]. The World Health Organization has also announced that the scope of pharmacovigilance is expanding due to globalization, consumerism, the explosion of free trade, international communication, and the increasing use of the Internet. In the future, the scope of pharmacovigilance may extend from drugs to herbal medicines, traditional medicines, alternative medicines, nutraceuticals or cosmetics and medical devices; it will not be limited to the monitoring of adverse reactions and events but will evolve to focus on new issues and challenges, such as the emergence of illegal sales under e-commerce and the potential risks or adverse effects of traditional medicines. Likewise, the formulation of pharmacovigilance should be more standardized; for example, current adverse reactions and precautions in many drug instructions are still “not yet clear” [38]. These need to be further standardized in the future development of pharmacovigilance under the premise of scientifically grasp its meaning.

Secondly, we should improve the pharmacovigilance system and clarify the responsibilities and obligations of all parties. Pharmacovigilance covers the whole life cycle of drugs in terms of time, and involves the safety, efficacy and economy of drugs [39]. In the whole process of pharmacovigilance work, it involves R&D, operation and use [31]. In the process of pharmacovigilance, it involves multiple organizations and departments, so the establishment of a sound organizational management system is the basis for further development of pharmacovigilance. First, it is necessary to promote the reform of current institutions and the transformation of the functions of various departments to improve the efficiency of supervision; second, it is necessary to consider the new mode of drug sales under the current development of the Internet and to implement public opinion monitoring and punishment measures related to pharmacovigilance [32,40]. Secondly, it is necessary to consider the new mode of drug sales under the current development of the Internet and implement measures for monitoring public opinion and penalties regarding pharmacovigilance. Furthermore, the importance of the uniqueness of Chinese medicine, in addition to the general regulation, importantly involves the coordination of the agricultural and forestry sectors related to cultivation. Overall, it is particularly important to improve the pharmacovigilance system, clarify the responsibilities and obligations among various organizational departments, and establish an effective communication and coordination mechanism among one another to realize the risk management of pre- and post-marketing drugs and to do a good job of integrating all aspects of pharmacovigilance and the whole pharmacovigilance work.

Thirdly, we should unlock new programs for pharmacovigilance regulation as soon as possible. With the continuous development of China’s pharmacovigilance work, China’s adverse drug reaction detection database is also expanding, but at present, the detection of adverse drug reactions and adverse events is still mainly collected passively [33]. This also leads to incomplete data and other limitations; therefore, it is important to develop new programs to convert monitoring into active and more comprehensive drug safety information. Currently, developed countries or regions such as Europe, Japan and the United States have been actively monitoring drug safety signals for many years [41]. For example, the U.S. FDA is working on a project called the “Drug Safety Monitoring Program”. For example, the U.S. FDA is working on an active pharmacovigilance system based on electronic medical records called “Sentinel Meter”, which is a distributed database that can improve the efficiency of drug safety-related data storage, management and sharing, as well as data mining and decision analysis with the help of third parties [42]. The program’s distributed database will improve the efficiency of storing, managing, and sharing data related to drug safety, and will enable data mining and decision analysis with the help of third parties. According to the 13th Five-Year Plan, China’s drug regulatory authorities have also launched the construction of sentinel hospitals for adverse drug reaction monitoring [33] and China’s hospital pharmacovigilance system, but only data collection has been achieved so far [41], as further data analysis management can learn from international experience, further improving its strength.

Similarly, with the rapid development of social media and big data, studies have proposed a framework for future adverse drug reaction monitoring through social media, where data information from different sources is collected and categorized and integrated [43]. Subsequent consideration is given to the possibility of applying artificial intelligence to identify safety risks from the collected data and unlocking new ways of pharmacovigilance regulation with the help of the Internet and media.

Fourthly, it is very important to build a pharmacovigilance system for Chinese medicines that is in line with their characteristics. Chinese medicine and proprietary Chinese medicines play a crucial role in the prevention and treatment of novel coronavirus pneumonia. The regulation of TCM is different from that of chemical drugs and biological products, which requires quality control in many aspects such as cultivation, harvesting, preparation, storage, toxicity, and compounding [44]. It is difficult to achieve the whole life cycle regulation of TCM. Although China has conducted many studies related to pharmacovigilance of TCM, such as the systematic evaluation of the safety of TCM, an analysis of case reports of adverse reactions of TCM, and an establishment of an ADR warning model of TCM, the establishment of a pharmacovigilance system of TCM is still in the exploration stage. The construction of a pharmacovigilance system for TCM that meets the characteristics of TCM will provide a reference for international pharmacovigilance regulation of herbal medicines and botanicals.

Fifthly, we need to strengthen international cooperation to develop a quality of pharmacovigilance that is internationally aligned. The EU pharmacovigilance system, the WHO Uppsala Testing Center system, and the ICH system, as the top three internationally recognized pharmacovigilance systems, each have their own characteristics. In recent years, China has attached great importance to international exchanges with the WHO, Uppsala Testing Center, ICH and other countries’ regulatory agencies, and actively held and participated in academic exchanges on pharmacovigilance so that international exchanges have gradually deepened. Further international integration with the WHO Uppsala Testing Center, ICH and other international organizations will further strengthen exchanges and communication. At the same time, we also cooperate with international herbal medicine monitoring organizations such as the WHO International Herbal Medicine Regulatory Cooperation (IRCH) and the Western Pacific Herbal Medicine Regulatory Forum (FHH) to carry out vigilance regulation of traditional medicines and improve the international level of Chinese medicine regulation.

**Figure 2:**
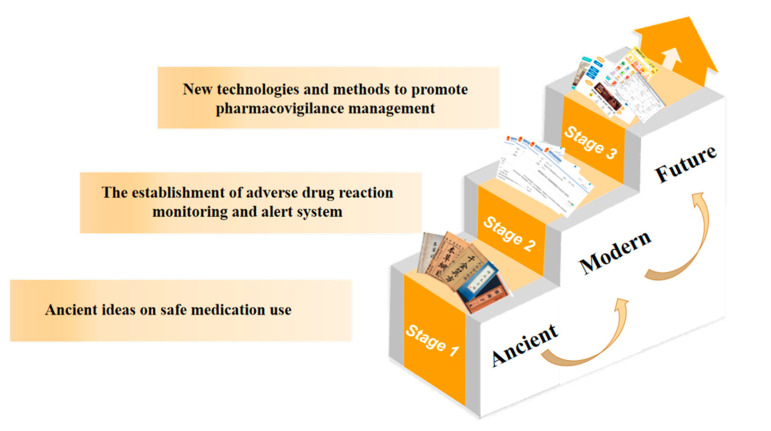
Brief historical development chart of pharmacovigilance of Chinese medicine.

Sixthly, it is imperative to strengthen education in pharmacovigilance thinking and to raise awareness of pharmacovigilance among the entire population. With the increasing youthfulness of the current society, high incidence of chronic diseases, and easy availability of drugs, pharmacovigilance is playing an increasingly important role in people’s medical lives. Especially in the case of the new epidemic, when everyone is facing the critical moment of “fighting” against the virus, choosing the right drugs and using them appropriately becomes the key to defeating the virus. It is not uncommon for the news to show that the combination of drugs and unreasonable use of drugs leads to other diseases, which fully illustrates the lack of awareness of pharmacovigilance among all people, even in urban areas, not to mention poor areas with poor medical standards. In addition to evaluating the efficacy, safety, and economy of drugs throughout their life cycle, pharmacovigilance also needs to popularize drug-related knowledge among the public and improve public health rights [37]. Therefore, it is imperative to narrow the gap between urban and rural areas, improve rural medical care, strengthen the ideological education of pharmacovigilance for the whole population, cover the whole country with pharmacovigilance awareness, and raise the awareness of safe drug use for the whole population. The above can be summarized in Figure 2.

## 6. Summary

Pharmacovigilance plays an important role in the entire life cycle of drugs, and a strong and perfect pharmacovigilance system is needed to regulate the entire life cycle of drugs, as well as for all participants in society to carry out active monitoring, build a social pharmacovigilance pattern, and apply risk management to all aspects of drug development, production, operation, use, and post-marketing management. In the future, a more systematic pharmacovigilance regulatory system is needed to provide support for vigilance activities, promote a smooth interface between the two regulatory phases of pre-marketing and post-marketing of drugs, and avoid regulatory dead ends. At the same time, pharmacovigilance in future development should be in line with China’s national conditions and characteristics, dovetail with international standards, highlight the characteristics of the body, and build a pharmacovigilance system with Chinese characteristics.

## Figures and Tables

**Table 1:** Table of records of major chronological works.

Dynasties	Publications	Main Ideas
Western Zhou Dynasty	*Zhou Li–Tian Guan–Tsukazai* (1046–771 BC)	The use of “poisons” to cure diseases; it is recorded that “the physician is in charge of the medical government and gathers poisons for medical services
Qin and Han Dynasty	*The Yellow Emperor’s Canon Internal Medicine* (475 BC to 220 AD)	The germ of the traditional Chinese idea of drug vigilance, the first classification of drugs into toxic and non-toxic
Western Han Dynasty	*Huainanzi-Xiuwu Xun* (1st century AD)	The earliest knowledge of the safety of Chinese medicine, there is a record of “one day and encounter 70 poisons”
Eastern Han Dynasty	*Shen Nong’s Herbal* (1st century AD)	The idea of compounding to reduce toxicity was first proposed, laying the foundation for the establishment of a pharmacovigilance system for Chinese medicine
	*Treatise on Cold-Induced and Miscellaneous Diseases* (200–210 AD)	Early drug cautionary ideas such as “mistreatment”, “change of evidence”, “bad disease” and “contraindications to treatment” were proposed
	*Synopsis of Prescriptions of the Golden Chamber* (Early 3rd century AD)	Documented dietary contraindications and drug dose control principles after taking drugs
Wei and Jin Dynasty	*Miscellaneous Records of Famous Physicians* (3rd–4th century AD)	The first classification of toxic drugs into the three grades of major, toxic and minor toxicity marked the emergence of the idea of grading the toxicity of Chinese medicine.
Eastern Jin Dynasty	*A Handbook of Prescriptions for Emergencies* (3rd century AD)	China’s earliest work on the methods of detoxification of traditional Chinese medicine, laying the foundation for the idea of detoxification of traditional Chinese medicine
Northern and Southern Dynasties	*Collection of Notes on the Materia Medica* (480 AD)	For the first time, drug warnings such as “fear of evil and contraindications” and “contraindications to taking medication and food” were systematically compiled and further elaborated on as regards the dosage of medication.
Tang Dynasty	*Invaluable Prescriptions for E-mergencies* (652 AD)	A special section is devoted to recovery from drug poisoning
	*Supplement to the Invaluable Prescriptions* (682 AD)	Summarizes the adverse reactions due to toxicity for each type of drug
	*A Supplement to Compendium of Materia Medica* (739 AD)	
Song Dynasty	*Classic Classified Materia Medica for Emergency* (1086 AD)	Summarizes the drug warning ideas in the herbal works of the past generations; adds toxic drugs; toxicity is divided into four levels: major, toxic, minor and slightly toxic; and for the first time set up a category of “poisonous herbs”
	*A Precious Manual of Obstetrics for Home Use* (992 AD)	Contained the first song on pregnancy contraindications
	*Taiping Shenghui Fang–Vol. 39–Prescriptions for Poisoning of Various Medicines* (1117 AD)	A detailed description of drug poisoning rescue is given
	*Complete Record of Holy Benevolence—Miscellaneous Therapies* (1184 AD)	
Ming Dynasty	*Compendium of Materia Medic* (1578 AD)	Summarized drug warning ideas in the herbal medicine of the past generations; added toxic drugs; added the four levels of major toxicity, toxic, minor toxicity, slightly toxic; introduced the first “poisonous herbs” category
Qing Dynasty	*The Materia Medica Easy to Read* (1694 AD)	Refined the classification of toxic drugs
	*The Harmful Effects of Materia Medica* (1862 AD)	Concentrates the essence of our traditional pharmacovigilance thinking and further develops and enriches it

## Data Availability

No new data were created or analyzed in this study. Data sharing is not applicable to this article.
